# Evidence of crop production losses in West Africa due to historical global warming in two crop models

**DOI:** 10.1038/s41598-019-49167-0

**Published:** 2019-09-06

**Authors:** Benjamin Sultan, Dimitri Defrance, Toshichika Iizumi

**Affiliations:** 1 ESPACE-DEV, Univ Montpellier, IRD, Univ Guyane, Univ Reunion, Univ Antilles, Univ Avignon, Maison de la Télédétection, 500 rue Jean-François Breton, F-34093 Montpellier, Cedex France; 2 Institute for Agro-Environmental Sciences, National Agriculture and Food Research Organization, 3-1-3 Kannondai, Tsukuba, Ibaraki 305-8604 Japan

**Keywords:** Attribution, Climate-change impacts

## Abstract

Achieving food security goals in West Africa will depend on the capacity of the agricultural sector to feed the rapidly growing population and to moderate the adverse impacts of climate change. Indeed, a number of studies anticipate a reduction of the crop yield of the main staple food crops in the region in the coming decades due to global warming. Here, we found that crop production might have already been affected by climate change, with significant yield losses estimated in the historical past. We used a large ensemble of historical climate simulations derived from an atmospheric general circulation model and two process-based crop models, SARRA-H and CYGMA, to evaluate the effects of historical climate change on crop production in West Africa. We generated two ensembles of 100 historical simulations of yields of sorghum and millet corresponding to two climate conditions for each crop model. One ensemble is based on a realistic simulation of the actual climate, while the other is based on a climate simulation that does not account for human influences on climate systems (that is, the non-warming counterfactual climate condition). We found that the last simulated decade, 2000–2009, is approximately 1 °C warmer in West Africa in the ensemble accounting for human influences on climate, with more frequent heat and rainfall extremes. These altered climate conditions have led to regional average yield reductions of 10–20% for millet and 5–15% for sorghum in the two crop models. We found that the average annual production losses across West Africa in 2000–2009 associated with historical climate change, relative to a non-warming counterfactual condition (that is, pre-industrial climate), accounted for 2.33–4.02 billion USD for millet and 0.73–2.17 billion USD for sorghum. The estimates of production losses presented here can be a basis for the loss and damage associated with climate change to date and useful in estimating the costs of the adaptation of crop production systems in the region.

## Introduction

Accumulated evidence indicates that agricultural production is being affected by climate change^1^. Climate change poses an additional burden, especially for developing countries, in achieving food security goals when the population is rapidly increasing; therefore, yield improvement is necessary to meet the increasing demand for food. Food security is a high-priority development goal for national governments in food insecure regions, such as West Africa, as well as for the United Nations (as declared in Goal 2 of the Sustainable Development Goals). However, in the recent literature on the consequences of an increase in greenhouse gas emissions and associated climate change on crop yields in West Africa^2–5^, several studies estimate possible crop yield losses with adverse impacts on food security in the next decades, although the extent of these losses remains uncertain^2–4^. Two meta-analyses estimate that climate change will lead to a mean yield reduction of −8% in all Africa^3^ by the 2050 s and −11% in West Africa^4^ without adaptation. Thus, in those regions, agricultural investments not only in conventional high-yielding technology but also in adaptation are desired to achieve the development goal under climate change^6^.

Various possible adaptation options and their uncertainties have been assessed in the literature^2,7,8^. Increasing crop tolerance to high temperatures during the flowering period^7,8^ and increasing cultivars’ thermal time requirement^8,9^ have been found to be the most promising technical options amongst a large variety of realistic adaptation options for the production of sorghum and millet (late sowing, increased planting density, fertilizer use and water harvesting and use of cultivars with larger thermal time requirements and greater resilience to heat stress). However, we lack knowledge on the crop production losses induced by climate change, which is a basis for exploring how much adaptation of production systems to specific crop costs, although some estimates of aggregated adaptation costs for agriculture at the continental or global level are available^10,11^. More specific estimates of such losses are useful to lead to a better understanding of the relative importance of adaptation to other political issues and are crucial for the optimal prioritization of adaptation investments for supporting adaptation strategies in West Africa that may counteract the adverse effects of climate change.

This study aims to estimate the average crop production losses of millet and sorghum, two major crops in West Africa, due to human-induced climate change during the last decade 2000–2009. Since the potentially adverse effects of climatic changes are superimposed on top of high natural climate variability and technical and crop management improvements, we design a modelling framework that is able to estimate the historical yield without accounting for human influence on climate, and we compare this estimate to a more realistic climate simulation including both natural forcing and human activities. We investigate the robustness of the changes in terms of regional climate and crop yields relative to these non-warming counterfactual climate conditions using a large ensemble of 100 climate simulations to sample the internal variability of climate and two process-based crop models to quantify the uncertainty in the response of crop yield to climate change. Crop price data are then used to quantify the financial production losses due to human-induced climate warming. It is expected that our estimates can offer a sound basis to depict a more specific view of the adaptation costs in crop production systems in that region.

In the next section, we introduce the experimental setup, the bias-corrected ensemble simulation of a climate model (MRI-AGCM3.2) and the two crop models (SARRA-H and CYGMA) used in this study. In section 3, we analyse the simulations by separating (i) the impacts of historical human activities on the regional climate of West Africa and (ii) the impacts on crop yields due to historical climate change. Finally, in section 4, we summarize and discuss our results.

## Materials and Methods

### Factual and non-warming counterfactual climate simulations and historical climate data

The bias-corrected 0.5° daily factual and counterfactual non-warming ensemble climate simulation data described in Iizumi *et al*.^12^ were used to investigate the impacts of human activities on historical climate and agriculture in West Africa. The original climate model simulations of factual and counterfactual climate conditions for the period 1951–2010 are carried out using the Meteorological Research Institute Atmospheric General Circulation Model, version 3.2 (MRI-AGCM3.2) with a grid interval of 60 km^13^, and the results attributing the simulated changes in temperature and precipitation to human activities are presented in Shiogama *et al*.^14^, Imada *et al*.^15^ and Mizuta *et al*.^13^. The factual climate simulation represents actual climate conditions that are influenced by both human activities and natural forcing. Observed changes in sea surface temperature, sea ice, greenhouse gas concentration, ozone, anthropogenic aerosol burdens (sulfate, black carbon and organic carbon), volcanic sulfate aerosol loading and natural aerosol loading (sea salt and dust) are considered in the factual climate simulation. Variations in solar irradiance are not considered in the simulation. The non-warming counterfactual climate simulation represents a pre-industrial climate that lacks appreciable human influences on global climate systems. The sea surface temperature and sea ice are both detrended, as are greenhouse gas concentration in 1850, anthropogenic aerosol and volcanic sulfate aerosol in 1850 and ozone concentration in 1961, which are used in the counterfactual climate simulation. Each type of climate simulation has 100 ensemble members associated with small perturbations in sea surface temperature that represent observational uncertainties. Additional details on the setup of the climate model and simulations are available in Mizuta *et al*.^13^, Shiogama *et al*.^14^ and Imada *et al*.^15^.

Observed monthly values of rainfall and temperature from the Climate Research Unit^16^ (CRU) are used to compare the accordance between the observations and the factual climate simulations, and then, the counterfactual climate simulations are characterized relative to the factual climate simulations. In addition, the global retrospective 0.5°-resolution 56-year (1958–2013) daily meteorological forcing data set, referred to as S14FD^17^, a hybrid of JRA-55 Japanese reanalysis data and gridded observations, is used as inputs of the crop models to simulate historical yields.

### Climate indices

To investigate the impacts of historical human activities on the regional climate of West Africa, several user-relevant indices for agriculture from the AMMA-2050 project^18^ were calculated on every land point of each factual and counterfactual ensemble climate simulation (Table 1). These include seasonal characteristics of precipitation (annual amount, number of rainy days, rainfall intensity, onset of the rains), mean surface temperature and extreme indices with heavy precipitation indices and one hot day index. Each index is averaged across West Africa (20°W to 15°E; 4°N to 18°N). The robustness of the change of an index between the factual and counterfactual climate simulations, on the basis of the models’ ensemble, is assessed with statistical tests. First, we average each of the 100 members of the factual and counterfactual ensemble climate simulations and test the statistical significance of the change between the two ensemble means over the last decade of the simulation 2000–2009 by means of Student’s t-test. Second, we apply a similar Student’s t-test to compare the factual and counterfactual climate simulations over the reference period 2000–2009, but for each model run individually, paired with the same initial conditions, and we count the number of significant changes at the 5% confidence level. A value of 100 in a climatic index of interest indicates that the factual and counterfactual climate simulations are significantly different in the 100 simulations. Third, we apply a two-sample Kolmogorov-Smirnov test to compare the 100 factual and 100 counterfactual climate simulations over the reference period 2000–2009 with the null hypothesis that the discrepancies between the two distributions are only due to sampling error. A significance level of 5% indicates that the null hypothesis can be rejected statistically. For Student’s t-test, we count the number of significant changes at the 5% confidence level. Finally, we count the number of cases with consistent change over the reference period 2000–2009 in a climatic index of interest between an ensemble member and the ensemble mean in terms of the sign of change. A value of 100 indicates that all changes in individual runs have the same sign as the change computed with the ensemble mean.

**Table 1:** User-relevant indices of climate for agriculture applications in West Africa.

User-relevant Indices	Description
Annual Mean Temperature	Annual mean surface temperature (°C)
Annual Rainfall	Total rainfall amount per year (mm/year)
Annual Rainy Days	Number of days per year with rainfall above 1 mm
Heavy Rainfall Events	Number of days per year with rainfall exceeding 30 mm/day
Very Heavy Rainfall Events	Number of days per year with rainfall exceeding 50 mm/day
Rainfall Intensity	Ratio between total annual rainfall and the number of rainy days per year
Very Hot Days	Number of days per year with daily mean surface temperature exceeding 30 °C
Onset Day	Onset of the rains computed as in *Bombardi RJ*, *Pegion*, *KV*,*Kinter JL*, *Cash BA and JM Adams (2017) Sub-seasonal predictability of the onset and demise of the rainy season over monsoonal regions*, *Frontiers in Earth Science 5*, *14*.

### Crop models

Sorghum and millet are the two main staple crops of sub-Saharan West Africa (59% of the total cereal production over the 2000–2009 period, on average; FAOSTAT data). The annual yields of millet and sorghum derived from two different process-based crop models were used. One was SARRA-H^9^, a model with a detailed process-based approach for phenology and carbon assimilation, and another was the CYGMA model^6^, with less details on water and carbon processes but including technological improvements. Using these two models with different modelling concepts under similar climate change conditions allows assessment of the uncertainty of the simulated yield response, as different parameterizations of physical and biological processes in crop models play a critical role in assessing and simulating yield responses to future climate change and adaptations^6,19,20^. Indeed, a similar change in terms of temperature, rainfall and/or radiation is likely to affect crop yields differently in the two models (Table 2); thus, consensus in the sign or in the amplitude of climate change may strengthen the confidence of the results.

**Table 2:** Summary of the main processes affecting crop yields under climate change simulations framework in the two crop models SARRA-H and CYGMA. Details on the processes can be found in Kouressy *et al*.^22^ and Iizumi and *et al.*^6^, respectively.

SARRA-H	CYGMA
Temperature acts on plant development rates through the concept of thermal time	Crop development is modelled using the growing degree days. The crop thermal requirement varies by crop and location.
Temperature has an effect on maintenance respiration	Temperature affects potential and actual evapotranspiration rates. The daily increment of leaf area and yield are stressed when the actual evapotranspiration is lower than its potential value.
No cold/heat stress	Each of heat and cold stresses are modelled as a function of daily mean temperature and used to compute the daily actual increment of leaf area and yield.
Water deficit affects plant transpiration and carbon assimilation	Water deficit defined as a ratio between actual and potential evapotranspiration rate is used to compute the daily actual increment of leaf area and yield.
Rainfall triggers sowing dates, and water stress can lead to sowing failures	Sowing date is determined according to 10-yr average temperature and moisture conditions. Sowing date is updated every year in the model. A crop-specific look-up table is used to derive the sowing date under a given climate condition.
No nitrogen stress	Stress associated with nitrogen deficit is modelled as a function of annual nitrogen application rate.
Solar radiation impacts potential assimilation rates through the intercepted, photosynthetically active radiation	Solar radiation affects daily potential increment of crop total biomass. After the modelled heading date, the CYGMA model starts calculating the daily increment of yield based on the intercepted photosynthetically active radiation, CO_2_ fertilization and fraction of the increment of the total biomass allocated to the harvestable part of a crop.
No CO_2_ effect	The CO_2_ fertilization effect is considered. The radiation-use efficiency changes depending on CO_2_ concentration.
Cultivar choice	The total thermal requirement of a crop that represents the cultivar’s characteristics on crop duration changes depending on the 10-yr average temperature condition and is updated every year in the model.
No technology improvements	The nitrogen application rate increases with changes in per capita GDP and per capita agricultural area. The crop’s tolerance to suboptimal conditions (nitrogen deficit, heat, cold, water deficit, and water excess) increases with changes in per capita GDP, GDP share of agriculture value added and total R&D expenditure (these are used to calculate agricultural knowledge stock), which represents the use of high-yielding technology and management.
Only rainfed condition is assumed	Yields in rainfed and irrigated conditions are separately simulated, and their area-weighted average is then computed. Because there is almost no irrigated area in West Africa, the millet and sorghum yields in West Africa simulated by the CYGMA model represent those in the rainfed condition.

The SARRA-H model was originally developed to simulate crops in dry tropics, such as millet and sorghum^21^. The inputs to the model are daily weather data, latitude, soil characteristics (depth, soil water capacity) and crop management (sowing density and depth, sowing date). The crop model estimates attainable yield under water-limited conditions by computing the soil water balance, evapotranspiration, phenology, assimilation, and biomass partitioning (see ref.^22^ for a detailed review of the model concepts). The water balance simulates runoff using a rain event empirical threshold of 20 mm^23^, soil evaporation, storage, deep drainage, transpiration and soil surface evaporation. Water stress is evaluated from the fraction of transpirable soil water according to Sinclair and Ludlow^24^, which acts as a reducing factor on plant transpiration and carbon assimilation based on FAO guidelines. Carbon assimilation and partitioning is explicitly simulated in the model^20^ with assimilation rates depending on solar radiation and water stress and with maintenance respiration depending on temperature. Daily temperatures are also involved in the computation of thermal time, which serves, on an additive basis, to calculate the progress of developmental processes. The phenology of the model is based on a simplified concept of successive phenological stages that have constant (genotypic) thermal duration. The photoperiod sensitivity routine is not activated in this study. SARRA-H was calibrated against local agronomic-trial data and on-farm surveys conducted in eight contrasted sites in terms of climate and agricultural practices in West Africa^25^. These data were used to define the varieties and the parameters representing management practice in the crop model. SARRA-H showed good performance in simulating crop yield variability in West Africa compared to FAO data, even if it tends to overestimate the mean yield^9^.

In contrast, the CYGMA model is a global gridded crop model and initially covers four major crops (maize, soybean, rice and spring wheat); it is modified in this study to include millet and sorghum. In the CYGMA model, the development of the crop growth stage is computed as a fraction of the accumulated growing degree days relative to the crop thermal requirements. Leaf area expansion and senescence are computed according to the fraction of the growing season using the prescribed shape of the leaf area index curve. The yields are computed from the photosynthetically active radiation intercepted by the crop canopy, the radiation-use efficiency, the CO_2_ fertilization on the efficiency and the fraction of total biomass increments allocated to the harvestable component. The soil water balance submodel and the snow cover submodel are used to calculate the actual evapotranspiration (although no snow cover occurs in the studied area). Five different stress types – nitrogen (N) deficits, heat, cold, water deficits and water excesses – are considered. The most dominant stress type for a day decreases the daily potential increment of the leaf area and yield. All of the stress types except N deficits are functions of daily weather conditions. The stress associated with the N deficit is modelled as a function of the annual N application rate. The crop’s tolerance to these stresses increases as the knowledge stock increases. The knowledge stock is an economic indicator derived as the sum of the governmental annual research and development (R&D) expenditures in the agricultural sector since 1961 with a certain obsolescence rate, and it represents the average agronomic technology and management level among farmers in the country. More details on modelling are available in the Supplementary Note described in the Supplementary Information of Iizumi *et al*.^6^.

The CYGMA model uses socioeconomic variables (such as per capita GDP, per capita agricultural area, agricultural research and development expenditure) as the model inputs in addition to physical variables (e.g., daily weather and plant-extractable water holding capacity of soil), enabling the model to simulate yield trends associated with technological improvements. This is a unique characteristic of the CYGMA model. The daily mean, maximum and minimum temperatures, precipitation, solar radiation, relative humidity and wind speed are used as the weather inputs. For this study, the CYGMA model was calibrated for millet and sorghum using the global dataset of actual and potential yields in 2000^26,27^, as was done for the four crops in earlier work^6^. In short, the sensitivity of the daily increment of leaf area index and yield to each of five different stress factors (N deficit, heat, cold, water deficit and water excess) was determined using the relationship among the yield gap (the ratio between actual and potential yields) in 2000, knowledge stock in 2000 and simulated average growing season stress factor in 1996–2005. The crop coefficient values for millet and sorghum used in this study are available in Table S1. The CYGMA model operates globally for millet and sorghum, similar to the four crops previously modelled; therefore, the simulated yields in West Africa were used for this study.

### Historical, factual and counterfactual crop simulations

The crop model simulation experiment has two purposes (Table 3). First, climate observation data are used to force the crop models to compare simulated crop yields with observed crop yields and check the performance of SARRA-H and CYGMA on the historical period. The two crop models are run using the same historical forcing dataset S14FD in West Africa (20°W to 15°E; 4°N to 18°N) for the period 1961–2012. Second, factual and counterfactual climate simulations are used to force the crop models to reproduce the crop yields under climate with (factual) and without (counterfactual) human influences. The comparison between the factual and counterfactual crop yield simulations enables the quantification of the effect of human influence on historical crop yields as simulated by the two crop models.

**Table 3:** The crop simulation design.

Climate forcing dataset	CO_2_	Crop model	Purpose of simulations
S14FD retrospective meteorological forcing dataset^17^	Historical (317 ppm in 1961 to 389 ppm in 2010)	CYGMA and SARRA-H	Comparison between simulated crop yield and reported yield data
Bias-corrected MRI-AGCM3.2 simulations with human influence on climate systems^13–15^			Factual crop yield simulation
Bias-corrected MRI-AGCM3.2 simulations without human influence on climate systems^13–15^	287 ppm (the level of the year 1850)		Counterfactual crop yield simulation

The SARRA-H simulations include four sets of parameters to capture the diversity of crop management in West Africa. They aim to reproduce the behaviour of two representative varieties of millet and two varieties of sorghum that differ mainly by the length of the growth cycle– a short duration crop (approximately 90 days) and a long duration crop (approximately 120 days) that are both insensitive to the photoperiod – and two levels of fertilization rate by modifying the biomass conversion ratio to a sub-optimal level and a better level to simulate, respectively, a low and a moderate fertilization rate. Here, we run the model separately using each of these four sets of parameters and average the resulting yield with even weights year by year, as in an earlier study^9^, since no information is available to determine weights for the individual management conditions. The available water holding capacity of the soil (between wilting point and field capacity) was set to 100 mm m^−1^, and the upper limit of the rooting depth was fixed at 1800 mm (see more details in ref.^9^). The sowing date was generated by the model and defined as the day when the simulated plant available water in the soil was greater than 10 mm at the end of the day, followed by a 20-day period during which crop establishment was monitored^28^. The juvenile stage of the crop was considered to have failed, triggering automatic re-sowing if the simulated daily total biomass decreased during 11 out of 20 days. Simulations were performed without any irrigation since most crop systems are rainfed (93% of all agricultural land in Sub-Saharan Africa; FAOSTAT data), and to our knowledge, irrigation is never used for pearl millet and sorghum in West Africa.

The CYGMA model required a 60-yr long soil moisture spin-up (1958–1960 × 20 times) before crop simulation. Annual nitrogen application rates for the individual crops were parameterized as a function of per capita GDP and per capita agricultural area, with the assumption that an increase in average farm income and an increase in land scarcity lead to increased fertilizer use. The use of improved varieties and corresponding management was parameterized using the agricultural knowledge stock, which is an economic indicator to represent technological improvements. These socioeconomic data were obtained from the World Bank and FAOSTAT databases. The sowing dates of the individual crops in 2000 in the CYGMA model follow those reported in the Monthly Irrigated and Rainfed Crop Areas around the year 2000 (MIRCA2000) dataset^29^ but change dynamically according to the long-term temperature and moisture conditions calculated in the model. The extent of harvested area and the fractions of irrigated and rainfed areas in 2000 were obtained from the M3-Crops dataset and MIRCA2000 dataset, respectively. We also used the Historical Irrigation Information (HID) dataset^30^ to take the annual growth rates of irrigation-equipped area (if any) into account. However, in West Africa, there are almost no irrigated areas. Therefore, the crop yields in West Africa simulated by the CYGMA model represent those in rainfed conditions.

Factual and non-warming counterfactual crop simulations were performed using the two crop models with the 100 factual and counterfactual ensemble climate simulations as the inputs to the crop models to estimate the impacts of historical climate change on crop yields. The climatic variables used as the model inputs include daily mean, maximum and minimum 2-m air temperatures, precipitation, downward shortwave radiation flux, relative humidity and 10-m wind speed. The ET_0_ estimation is calculated by the Penman-Monteith FAO56 equation^31^ in the SARRA-H model and a variant of this equation in the CYGMA model^6^.

### Production losses associated with historical climate change

For a given year, country and crop, we first calculated the yield impacts at a grid-cell level by comparing the historical and non-warming counterfactual crop simulations:

1 $$\Delta {Y}_{t,g}=({Y}_{historical,t,g}\,\mbox{--}\,{Y}_{counterfactual,t,g})/{Y}_{historical,2000:2009,g},$$

where suffix *t* and *g* indicate the year and grid cell, respectively; *ΔY* is the simulated grid-cell yield impact (ratio); *Y*_*historical*_ is the simulated grid-cell yield under the factual climate condition (t ha^−1^); *Y*_*counterfactual*_ is the simulated grid-cell yield under the counterfactual climate condition (t ha^−1^); and *Y*_*historical*, 2000*:2009*_ is the simulated grid-cell average yield over the baseline period (2000–2009) calculated using factual crop simulation data (t ha^−1^). The calculated grid-cell yield impacts in a country were aggregated:

2 $$\Delta {Y}_{t,c}=\frac{{\sum }_{g=1}^{G}\Delta {Y}_{t,g}\times {A}_{M3Crops,2000,g}}{{\sum }_{g=1}^{G}{A}_{M3Crops,2000,g}},$$

where suffix *c* indicates the country, and *A*_*M3Crops* 2000_ is the reported grid-cell harvested area in 2000 from the M3Crops dataset (ha). To avoid unrealistically large yield impacts, which could numerically occur when the simulated yield under the factual climate condition is low (e.g., <0.1 t ha^−1^), we replaced *ΔY*_*t*_,_*c*_ with −100% when *ΔY*_*t*_,_*c*_ was below −100%. In contrast, *ΔY*_*t*_,_*c*_ was replaced with +100% when it was greater than +100%. Finally, the production impact was calculated as follows:

3 $$\varDelta {P}_{t,c}=\varDelta {Y}_{t,c}\times {Y}_{FAO,2000:2009,c}\times {A}_{M3Crops,2000,c}\times {P}_{FAO,2000:2009,c},$$

where *ΔP* is the impact on annual production of a crop of interest on a country level (USD); *ΔY* is the simulated country annual yield impact relative to the simulated average factual yield in the baseline period (ratio); *Y*_*FAO*, *2000:2009*_ is the FAO-reported country average yield in the baseline period (t ha^−1^); *A*_*M3Crops*_, _*2000*_ is the reported country harvested area in 2000 calculated using M3Crops dataset (ha); and *P*_*FAO*, *2000:2009*_ is the FAO-reported country average producer prices in the baseline period (USD t^−1^). In the FAO database, no effective harvested area data were available for some years and countries in West Africa. Therefore, while time-constant, we used the grid-cell harvested area data obtained from the M3Crops dataset instead of FAO-reported data. Different price data (i.e., consumer price) were available in the FAO database, but consumer prices are strongly affected by variations in oil price and export bans in major food-exporting countries. Therefore, we used producer prices to minimize the impacts of these confounding factors on our estimates of crop production losses.

## Results

### Impacts of historical human activities on the regional climate of West Africa

**Figure 1:**
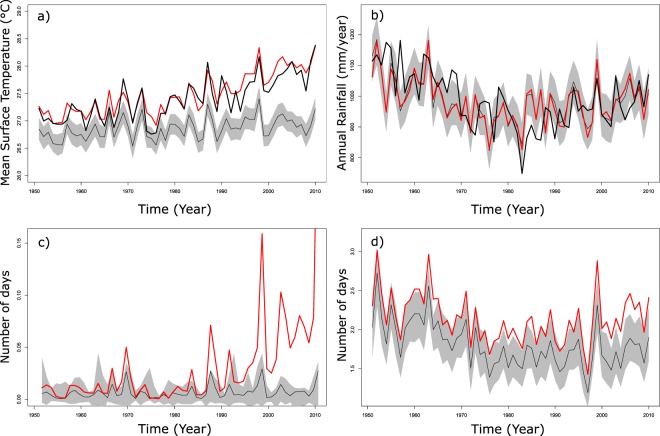
Ensemble mean of simulated counterfactual (thin black) plus standard deviation of the 100 members (grey envelope) and factual climate condition (red) of mean surface temperature (**a**), annual rainfall (**b**), number of very hot days (**c**) and number of very heavy rainy days (**d**). See Table 1 for the definition of the climate indices. The thick black line represents the observed annual mean temperature (**a**) and annual rainfall (**b**) from CRU data. All values are shown on average across West Africa (20°W to 15°E; 4°N to 18°N).

Human influence has strongly modified the annual mean temperature in West Africa, with a regional warming depicted since the 1950s (Fig. 1a). We found a difference of approximately 1 °C when comparing the average temperature of the last decade 2000–2009 between the factual and counterfactual climate simulations (Table 4). Although this warming is simulated all over West Africa, it is more pronounced in the north and central Sahel and in the Sahara (Fig. S1a). This is certainly the most robust change, with all statistical tests showing significant values (Table 4). In addition, the factual climate simulation, which includes human influence, is able to more accurately reproduce the mean and the variability of the observed annual temperatures derived from the CRU dataset^32^ than the counterfactual climate simulation (Fig. 1). The correlation between the observed and simulated time series of annual temperature reaches 0.92 with the factual simulation, while it falls to 0.75 with the counterfactual simulation. Moreover, the bias between the simulated and observed annual temperatures is −0.9 °C with the counterfactual simulation, which is colder than CRU data, while the bias is only +0.1 °C with the factual simulation. This comparison provides clear evidence of the reality of anthropogenic climate change in West Africa. The factual and counterfactual climate simulations significantly differ from each other. For instance, an important increase in the number of very hot days in the factual climate simulation was found (the number of hot days increases eight-fold in the last decade; Fig. 1c and Table 4). This increase is more pronounced in the north of West Africa (Fig. S1c). While the impacts of human activities are particularly evident on regional surface temperature, the impacts on rainfall are less clear. The model is able to accurately reproduce the decadal variations of annual rainfall with the wet years during the 1950s, the droughts of the late 1970s and 1980s and the recovery of rainfall, which starts in the 1990s compared to observations (Fig. 1b). However, the simulated annual rainfall time series in the factual and in the counterfactual simulations are almost identical, showing a similar correlation with observation (R = 0.54 and R = 0.56 for factual and counterfactual simulation, respectively) and no significant change in the last decade (Table 4). The changes are hardly or not significant when we consider the number of rainy days or the onset of the rains, which neither of which seems to be modified with the human activities in the climate model. However, more significant changes are detected for rainfall intensity and in heavy rainfall in the Sahel over the last decade, which can be deemed the impacts of human activities (Table 4; Fig. S1). This is particularly clear when we consider the number of rainy days exceeding 50 mm/day (Fig. 1d), where we found a significant increase of 27.7% in the last decades between the factual and the counterfactual simulations.

**Table 4:** User-relevant indices of climate in 2000–2009 with mean counterfactual climate condition and factual climate condition, differences between factual and counterfactual climate simulations (relative differences for all indices except for mean temperature and onset day). All values are shown on average across West Africa (20°W to 15°E; 4°N to 18°N). One (two) star(s) indicates that the difference in the ensemble mean is significant at the 1% (0.1) level. The three columns on the right indicate the number of the 100 runs that pass a Student’s test or a Kolmogorov test, which agree with the sign of the difference of the ensemble mean at the 5% significance level.

User-relevant Indices	Counterfactual	Factual	Difference	Student t-Test	KS Test	Sign Agreement
Annual Mean Temperature	26.9	28.0	1.0**	100	100	100
Annual Rainfall	974.8	983.9	0.9%	1	0	62
Annual Rainy Days	82.1	82.6	0.7%*	0	0	64
Heavy Rainfall Events	6.8	7.6	11.6%**	32	10	99
Very Heavy Rainfall Events	1.7	2.2	27.7%**	85	43	100
Rainfall Intensity	14.4	15.0	3.9%**	25	11	94
Very Hot Days	0.0	0.1	841.0%**	82	83	100
Onset Day	160.2	160.8	0.6*	1	0	66

### Performance of crop models to simulate historical yields

**Figure 2:**
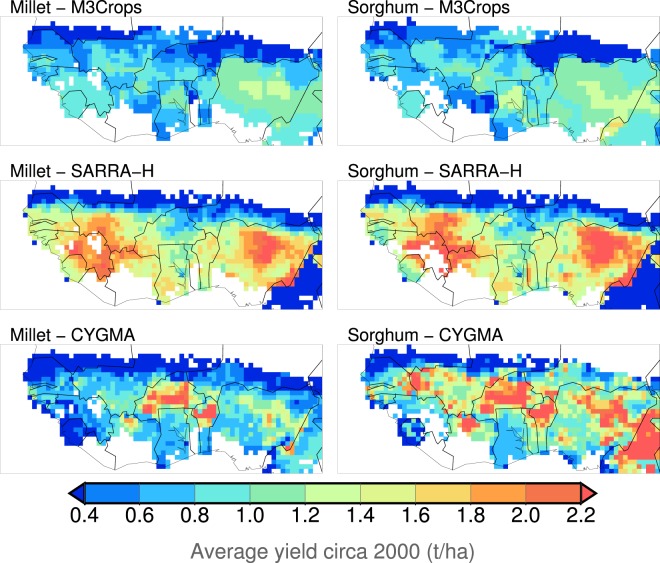
Geographical patterns of the reported and simulated average yields of millet and sorghum circa in 2000 across West Africa. The reported data were obtained from the M3Crops dataset^26^. Both crop models SARRA-H and CYGMA used the same forcing dataset S14FD. For SARRA-H outputs, only locations where the individual crops were harvested were shown. For consistent comparison with the M3Crop-reported data, the simulated annual yields for the period 1997–2003 were averaged and then compared.

The reported data for West Africa obtained from the M3-Crops dataset showed that the reported millet yields were almost the same as the sorghum yields (Fig. 2). The yield dataset is solely based on reported yield statistics and represents the average yield circa the year 2000 (that is, the average in 1997–2003). However, the different crop models reproduced the reported data differently. The average yields of millet in 1997–2003 simulated by the CYGMA model were comparable to the reported data in terms of the long-term yield average (the simulated yields are approximately 1 t ha^−1^), whereas the SARRA-H model overestimated the millet yields over that region (the simulated yields often exceed 2 t ha^−1^). Both crop models overestimated the sorghum yields, especially in northern Nigeria and southern Mali, while only the CYGMA model overestimated those in Burkina Faso.

**Figure 3:**
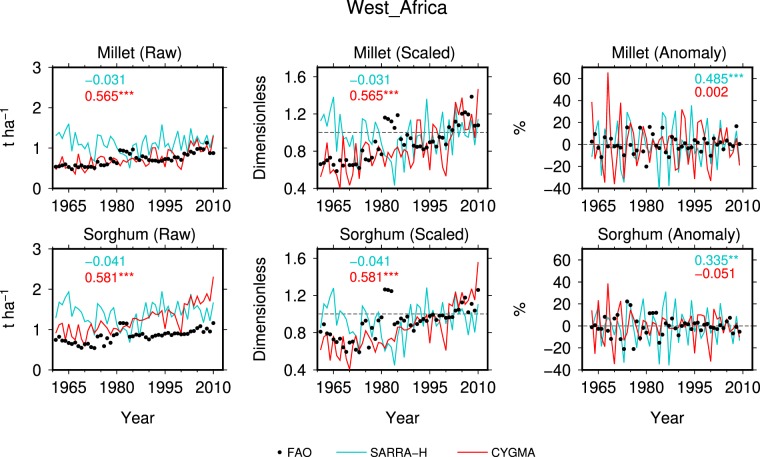
Comparison of annual yield time series reported by the FAO and the average yields over West Africa simulated by SARRA-H and CYGMA for millet and sorghum for the period 1961–2012. The regional average data for West Africa were calculated by averaging the data over Benin, Burkina Faso, the Gambia, Guinea, Guinea Bissau, Mali, Niger, Nigeria, Senegal, Sierra Leone and Togo (country harvested areas were used as the weights). The grid-cell harvested area in 2000 was used as the weight when computing country average yields. Three different metrics were used: 1) the reported and simulated raw yield data were compared without any post-processing (raw); 2) the reported and simulated annual yield data were separately scaled so that each average yield in 1996–2005 was equal to one, and then the data were compared (scaled); and 3) the percentage yield anomalies, relative to the normal yield calculated as the 5-yr running average, were separately computed for the reported and simulated data and then compared (anomaly). The numbers presented in each panel show the correlation coefficient calculated between the reported and simulated data (***, ** and * indicate that the correlation is significant at the 1%, 5% and 10% levels, respectively).

The CYGMA model showed a good match in terms of the regional average yield for millet relative to the reported data (“Raw” in Fig. 3), while the SARRA-H model led to a substantial overestimation. However, both crop models overestimated the regional average sorghum yields. Post-processing of the simulated yields, such as scaling so that the CYGMA-simulated regional average yield in 1996–2005 would equal one, led to a good agreement between the reported and simulated yields (“Scaled” in Fig. 3). These results indicate that the simulated regional average yield in absolute terms is less reliable and that the simulated relative changes in yields should be utilized for further analysis.

The main advantage of the CYGMA model is that it is able to reproduce the historical yield increasing trends, as visually indicated (“Raw” in Fig. 3). A sensitivity analysis using the CYGMA model revealed that the technological effect (increased use of N fertilizer and improved varieties along with an increase in the knowledge stock) contributed more strongly to the simulated yield trends than the CO_2_ fertilization effect (Fig. S2). No such trend is simulated with the SARRA-H model, which does not include technological effects nor the CO_2_ fertilization effect.

**Figure 4:**
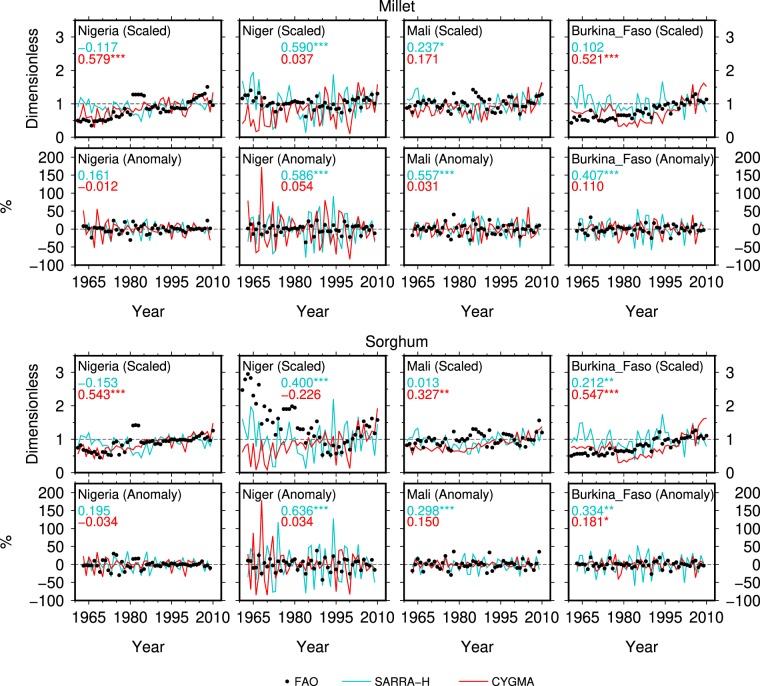
Same as Fig. 3, but for the selected major crop-producing countries in West Africa.

The SARRA-H model outperforms the CYGMA model in reproducing the interannual variability of crop yields. Significant correlation values between the reported and simulated regional average yield anomalies were obtained for both millet and sorghum (R = 0.485 and R = 0.335, respectively; “Anomaly” in Fig. 3). The country-level comparisons reveal that the performance of the SARRA-H model in simulating the yield anomalies of the two crops was relatively good, with significant correlation coefficients in Niger, Mali and Burkina Faso, three major crop-producing countries in the studied region (Fig. 4). The ability of the SARRA-H model to capture interannual variability is detailed for the 12 countries in Fig. S3. The highest correlation between simulated yield and reported yield is depicted for millet in Senegal (R = 0.77). However, the correlation coefficient was not significant for Nigeria, the top producer of sorghum and the 3^rd^ largest producer of millet in the region (Table S2). Therefore, if we remove the data in Nigeria when calculating regional average yield anomalies, the correlation values between the reported and simulated yields increase to 0.686 for millet and 0.533 for sorghum (figure not shows). The best correlations are obtained when rainfall and/or temperature during the growing seasons are correlated with the reported FAO yields (Fig. S3), i.e., when the observed yield variability is explained by climate factors. However, the SARRA-H model generally outperforms the correlation between the reported FAO yield and rainfall and/or temperature, which shows the added value of having a process-based model compared to simple linear statistics. The performance of the CYGMA model in simulating the yield anomalies of millet and sorghum was lower than that of the SARRA-H model in most cases.

These results suggested that the models evaluated here, although imperfect, had a certain usefulness and complementarity in assessing the climate impacts on yield variability and trends. The CYGMA model accurately reproduces the yield trends, while the SARRA-H model better reproduces year-to-year variability. However, the comparison between reported and simulated country yields in West Africa is difficult partly because – although FAO yield statistics are the best reliable source of information – many data values from the FAO database in the region are derived from data imputation or unofficial data sources (Table S2), and historical management information is not available.

### The impacts on crop production due to historical climate change

**Figure 5:**
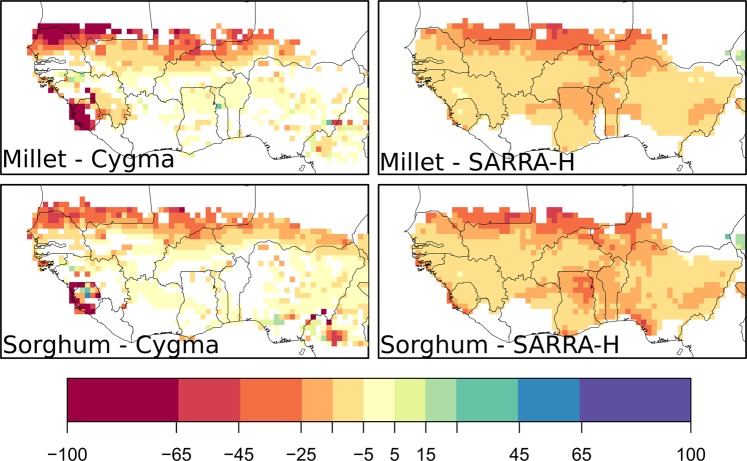
Geographical patterns of average yield impacts in 2000–2009 associated with historical climate change relative to a non-warming counterfactual climate condition. Areas in white indicate that the simulated yield change is non-significant at the 1% level.

The simulated impacts on average yields in 2000–2009 due to historical climate change varied by location and between the two crop models (Fig. 5). Although some positive impacts are simulated by the CYGMA model in the south of West Africa, negative yield impacts appeared in many parts of West Africa for both millet and sorghum. Both crop models simulate a strong crop yield loss in the northern part of the Sahel (north of 14°N), exceeding 50% in some locations. This tendency is common across millet and sorghum and consistent between CYGMA and SARRA-H.

**Figure 6:**
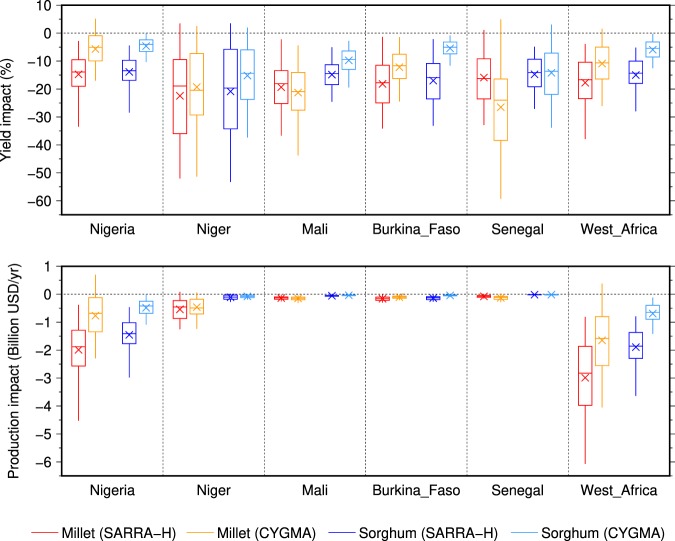
Estimated impacts on the average annual yield and average production of millet and sorghum in 2000–2009 for selected major crop-producing countries in West Africa. The impacts were measured as the difference between the factual and counterfactual crop simulations. Harvested area weighting was considered when computing country and West Africa average yield impacts. Box plots indicate the mean (cross), with 25 to 75% (box) and 5 to 95% (vertical line) confidence intervals derived from ensemble members of the crop simulation. The horizontal lines indicate the median. The regional average data for West Africa were calculated by averaging the data over Benin, Burkina Faso, the Gambia, Guinea, Guinea Bissau, Mali, Niger, Nigeria, Senegal, Sierra Leone and Togo. The range of simulated yield change was limited to the interval from −100% to +100% to avoid unrealistically large yield impacts in locations where the current yield is very low.

The estimated average yield impacts in the recent decade (2000–2009) due to historical climate change, relative to a counterfactual climate condition, appeared to be negative for all major crop-producing countries in West Africa (Fig. 6). Millet appears to be more affected by climate change than sorghum in the two crop models. On average, across West Africa, the SARRA-H model simulates a yield loss of 17.7% for millet and 15.0% for sorghum. The CYGMA model simulates a slightly weaker impact, with a yield loss of 10.9% for millet and 5.9% for sorghum. When we consider the five major crop-producing countries (Fig. 6), the highest production losses are estimated in Niger (sorghum), Senegal (millet) and Mali (millet). Important production losses are also expected in other countries, such as Benin and the Gambia (Fig. S4).

Using the grid-cell harvested area in 2000 and the country average producer prices in 2000–2009, we estimated that the average annual production losses over West Africa in 2000–2009 associated with historical climate change simulated by SARRA-H account for 2.99 billion USD for millet and 1.89 billion USD for sorghum (Fig. 6). These losses are lower when the CYGMA model was used and account for 1.65 billion USD for millet and 0.69 billion USD for sorghum. Although all of the simulations lead to economic losses in West Africa, the uncertainty persists. Using the CYGMA model, the 90% probability interval of the estimated sorghum production losses calculated across the 100 ensemble members, which represents the uncertainty of the internal climate variability, ranged from 0.12 to 1.42 billion USD. For millet, the estimates ranged from the production loss of 4.05 billon USD to the production gain of 0.38 billion USD. Using the SARRA-H model, the production loss estimates for sorghum varied from 3.64 to 0.79 billion USD for sorghum and from 6.07 to 0.81 billion USD for millet. The most important economic losses are expected in Nigeria and Niger partly because of the relatively large extent of their harvested area for millet and sorghum.

**Figure 7:**
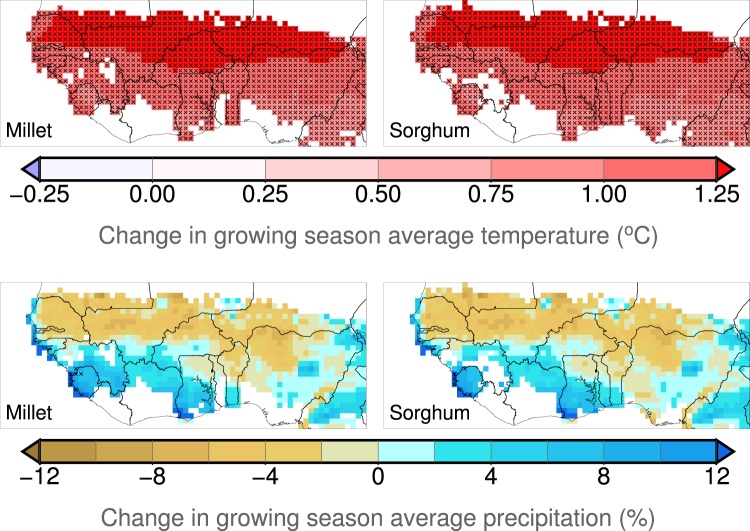
Geographical patterns of changes in growing season average temperature and precipitation in 2000–2009 relative to a counterfactual climate condition. A cross indicates that the change is significant at the 1% level. Areas in white indicate that a crop of interest is not harvested around the year 2000.

**Figure 8:**
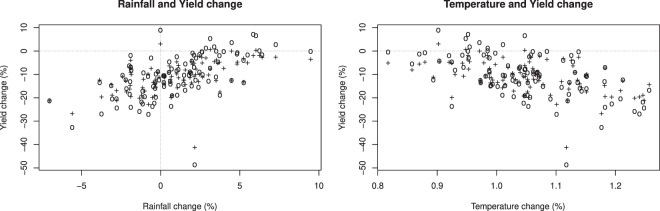
Relative yield changes of millet (o) and sorghum (+) simulated by SARRA-H in 2000–2009 relative to a counterfactual climate condition and relative changes of rainfall (left) and absolute change of mean surface temperature (right). All values are shown on average across West Africa (20°W to 15°E; 4°N to 18°N) for each of the 100 members of the ensemble simulation.

This production loss is mainly driven by the temperature change between the factual and the counterfactual climate simulations, whose spatial pattern (Fig. 7) is similar to the pattern of yield change (Fig. 5); the highest production losses are located in the northern Sahel, where the warming is more intense. The relationship between temperature change and yield change is particularly clear when the SARRA-H model is used (Fig. 8). A negative linear relationship is found between simulated yield change and temperature change. A warming above 1 °C is associated with yield losses in almost all simulations of millet and sorghum, and the warmest simulations lead to the highest yield losses. Although no significant change on simulated rainfall is found, on average, in West Africa, variations of rainfall can also have an impact on SARRA-H-simulated yield change and have the potential to exacerbate or mitigate the yield impact depending on whether the rainfall decreases or increases. However, there are only a few simulations showing production gain even with rainfall increase. This confirms the results of Salack^32^, who showed that rainfall increase did not completely compensate for the effect of temperature increase (+1.5 °C) but greatly mitigated the impacts of warming (−59% and −26% for decreasing and increasing rainfall, respectively). The relative effects of temperature and rainfall changes on crop yield impacts are also discussed by Roudier *et al*.^4^ and Schlenker and Lobell^33^, who also noted the dominant role of temperature increase in the estimation of the impact of climate change on crop yield in Africa. However, there are uncertainties in the estimated climate-yield relationship across the two models SARRA-H and CYGMA. Indeed, although the CYGMA-simulated yields decrease with warming and increase with increasing precipitation, the slope is weaker (Fig. S5), which might explain why the simulated yield impacts are lower in CYGMA than in SARRA-H.

To better understand the production changes in the processes represented in the crop models, a sensitivity analysis was performed with the CYGMA model, which simulates more processes affecting crop yields (Table 2) and has more flexibility. The results (Fig. S6) suggest that CO_2_ fertilization and technological improvement are insufficient to offset the increased heat stress and water stress under factual conditions. The water deficit stress most largely contributed to the yield impact, likely through increased evapotranspiration since no significant trend in growing season rainfall was detected (Fig. 7). A direct effect of warming, such as heat stress, is also found, but its contribution to the yield impact is smaller than that of the indirect effect. Interestingly, although the SARRA-H model includes fewer processes and does not include heat stress, it leads to crop yield losses similarly to CYGMA. This consistency might imply that mechanisms commonly considered in the two models cause yield and production losses. Increased evapotranspiration-led water deficit was found to be the largest contributing factor in CYGMA (Fig. S6) and in SARRA-H in a previous study^9^, in addition to shortened crop duration with increased temperatures. However, CYGMA-based yield and production losses are almost always smaller than the SARRA-H-based yield and losses (an exception is millet in Senegal). One explanation is that the SARRA-H model overestimates the effect of climate on crop yield compared to the CYGMA model or the reported yields from the FAO (Fig. S7) and thus might exacerbate the effect of altered climate conditions. In addition, technological effects (use of varieties that are more tolerant to suboptimal conditions and have larger thermal requirements) and CO_2_ fertilization effects are considered in CYGMA but not in SARRA-H and alter the yield response (Fig. S6).

## Discussion and Conclusions

Here, we used a large ensemble of climate simulations and two crop models to estimate the effects of historical climate change on crop production in West Africa. Our study demonstrates that human activities have already had a significant impact on the regional climate in the Sahel, with negative consequences on agricultural production.

The recent increase in the mean temperature in the Sahel and the Sahara, as observed in the CRU observations and described by several authors (see, for instance, ref.^34^), cannot be reproduced if the influences of human activities on climate are excluded. The decade 2000–2009 is approximately 1 °C warmer in West Africa, relative to a counterfactual climate condition, due to human activities, with a much higher frequency of very hot days. While the evolution of annual rainfall in West Africa during the 20th century, which is characterized by the succession of wet periods with droughts^35^, does not seem to be driven by human activities, we found a significant increase in the intensity of rainy events. This increase in the frequency of heavy rains corroborates the results from Taylor *et al*.^36^ and Panthou *et al*.^37^, who depicted more intense Sahelian storms since the 1980s using 35 years of satellite observations and rain gauges in the region. The authors also attributed this intensification of the rains to global warming, which particularly affects the temperatures in the Sahara. A warmer Sahara intensifies convection within Sahelian storms through increased wind shear and changes to the Saharan air layer^38^.

The two crop models used here differently reproduced the reported yields in West Africa. Although no single best crop model was found, the CYGMA model showed its relative advantage in capturing the yield growth driven by technological improvements. In contrast, the SARRA-H model showed its relative advantage in capturing yield anomalies, which are mainly driven by seasonal climate variability. The modelled yield response to growing season mean temperature and precipitation are largely different across the models in quantitative terms but similar in qualitative terms (Figs 8; S5 and S7). The crop models reveal a common tendency for warmer conditions to lead to yield losses and wetter conditions to lead to yield gains in the studied region. For this reason, the negative impacts of historical climate change, relative to a counterfactual climate condition, are derived from the crop models. The yield impacts simulated by the SARRA-H model are often more severe than those simulated by the CYGMA model (Fig. 5). The explicit consideration of technological improvements and agronomic adjustments (sowing date shift and cultivar switching) in the CYGMA model partially explains the difference.

Crop production losses were assessed by two independent crop models for the two main staple food crops of West Africa, millet and sorghum. We found that the average crop yield across West Africa for the decade 2000–2009 would have been 11% to 18% higher for millet and 6% to 15% higher for sorghum if human influence on climate was excluded. This yield impact is quite substantial, especially for millet, when compared to the year-to-year variability of observed crop yield in FAO data. For instance, the dramatic drought of 1982–1984 led to a yield loss of approximately 18% in West Africa for millet compared to the more humid year 1985. This yield loss, which led to one of the most severe food crises in the Sahel, is within the range of our estimation of the average climate change impact in the decade 2000–2009 relative to a counterfactual climate condition. Although the uncertainty is large, crop production losses due to climate change have likely led to important financial losses in several Sahelian countries. We estimated that in Niger, which is one of the poorest countries on the planet, the climate change impact on millet production cost between 0.47 and 0.54 billion USD per year during the decade 2000–2009, while the average GDP of the country was 3.4 billion USD during the same decade, according to the World Bank. The GDP share of agriculture in Niger for the period is approximately 35%, and millet is the main cereal that is most extensively cultivated in that country. We assumed that among other agricultural commodities, the production share of millet in agricultural GDP accounts for 50%. The ensemble average yield impact for millet ranged from −19% (CYGMA) to −22% (SARRA-H) relative to what would have occurred under the counterfactual climate condition. A simple multiplication (3.4 billion USD × 0.35 × 0.50 × 0.22) leads to a production loss of 0.13 billion USD. Although this value is much smaller than the crop model-based production loss estimates, these estimates match in order. We found that estimated yield impacts sometimes take a very large value when the yield in the factual climate condition is low (<0.1 t ha^−1^), and this could numerically lead to an unrealistically large production loss estimate. We therefore limit the simulated maximum yield impact to −100% of the yield in the factual climate condition to avoid this problem. There might be room to further refine the threshold value, but no information is currently available to determine a reasonable value. The production loss estimates derived from the CYGMA model are smaller than those derived from the SARRA-H model mainly because the yield response to temperature in the CYGMA model is more moderate than that in the SARRA-H model.

Both crop models simulated more important impacts in the north of the Sahel, where the warming is the highest; Niger, Senegal and Mali are most affected by climate change. This spatial pattern is consistent with the results of Roudier *et al*.^4^, who found that cropped areas in the Soudano-Sahelian zone are likely to be more affected by climate change than those located in the Guinean zone. The authors explain this spatial variability by a greater warming in climate change projections over continental Africa (particularly in the Sahel and the Sahara), while the temperatures of the Guinean zone, which are influenced by the Atlantic Ocean, are expected to increase more slowly. Our results also confirm the importance of increasing temperatures, which leads to crop yield losses, irrespective of rainfall changes. This adverse role of higher temperatures in shortening the crop duration, increasing evapotranspiration demand and reducing crop yields has been noted in several crop modelling studies focused on West Africa^4,9,33,38,39^. These studies found that potential wetter conditions or elevated CO_2_ concentrations hardly counteract the adverse effect of higher temperatures^35^, while dryer conditions persistently appear as a cause of severe yield losses^4,9,34,40^.

Our production loss estimates for millet and sorghum are consistent with the estimated crop yield losses under future climate change scenarios published in the literature^2–4,40^. Indeed, it is expected that global warming in the next decades will lead to adverse effects on agriculture, with a mean yield reduction of −8% identified in all Africa^3^ and −11% in West Africa^4^. The production losses are already significant at moderate levels of regional warming. Here, we found that even an increase of 1 °C can lead to yield losses compared with the yields in the non-warming counterfactual climate condition. Challinor *et al*.^40^ and Parkes *et al*.^7^ showed that a robust decrease of crop yield and an increase of crop failure in tropical regions^40^ and especially in West Africa^7^ are expected with an elevation of 1.5 °C of the global surface temperature above preindustrial levels. Since the most optimistic climate change scenarios do not lead to a warming below 1.5 °C, crop production losses in West Africa appear to be unavoidable without adaptation, and knowledge of the most effective adaptation methods becomes critical. Several adaptation methods that may counteract the adverse effects of climate change were investigated for millet and sorghum in West Africa by Parkes *et al*.^7^ and Guan *et al*.^8^, and a review can be found in Sultan and Gaetani^2^.

Every modelling study has its limitations, and we recognize a few caveats to the design of our simulation experiments. Caution is necessary when interpreting the crop simulation results presented here because the quality of the reported data in the studied region is not as high as that in developed countries, although the FAO statistical database used here is the most reliable, accessible source of information on crop yields. The differences between the reported and simulated data described earlier are mainly due to imperfect modelling and errors in model inputs (this is especially the case for the socioeconomic variables used as the inputs to the CYGMA model) and the assumptions used in the model setup (e.g., the assumptions on management levels used in the SARRA-H model). However, errors in reporting, inadequate sampling in census surveys and data imputation could be other reasons for the differences. Indeed, a notable percentage of the data in the studied region is based on the data imputation at the FAO, with unofficial or unavailable sources (Table S2). For this reason, we used the harvested area data from 2000 obtained from the M3-Crops dataset throughout the studied period. However, FAO data show that the harvested areas of these crops change over time (14.6 M ha to 13.7 M ha for millet and 12.0 M ha to 13.8 M ha for sorghum for the period 2000–2016), which may lead to different production loss estimates. However, crop-specific gridded historical harvested area data are not available.

In conclusion, our study demonstrates that human activities have already had a significant impact on the regional climate in the Sahel, with negative consequences for the production of major cereals in the region. Our production loss estimates offer a sound basis to depict a more specific view of the costs of adaptation and investments in adaptation in crop production systems in West Africa. These estimates can provide scientific input for national governments and international organizations at international climate negotiations.

## Supplementary information

Supplementary Information

## References

[1] Cramer, W. *et al*. *Climate Change 2014: Impacts*, *Adaptation*, *and Vulnerability*. *Part A: Global and Sectoral Aspects* (eds Field, C. B. *et al*.), 979–1037, Cambridge Univ. Press (2014).

[2] Sultan, B. & Gaetani, M. Agriculture in West Africa in the twenty-first century: climate change and impacts scenarios, and potential for adaptation. *Frontiers in Plant Science* **7**, art. 1262 [20 p.]. ISSN 1664-462X (2016). 10.3389/fpls.2016.01262 PMC5004487 27625660

[3] Knox J, Hess T, Daccache A, Wheeler T (2012). Climate change impacts on crop productivity in Africa and South Asia. Environ. Res. Lett..

[4] Roudier P, Sultan S, Quirion P, Berg A (2011). The impact of future climate change on West African crop yields: what does the recent literature say?. Glob. Environ. Change.

[5] Arndt C, Asante F, Thurlow J (2015). Implications of climate change for Ghana’s economy. Sustainability.

[6] Iizumi T (2017). Responses of crop yield growth to global temperature and socioeconomic changes. Scientific Reports.

[7] Parkes, B., Defrance, D., Sultan, B., Ciais, P. & Wang, X. H. Projected changes in crop yield mean and variability over West Africa in a world 1.5K warmer than the pre-industrial era. *Earth System Dynamics* **9**, 119–134. ISSN 2190-4979 (2018).

[8] Guan K, Sultan B, Biasutti M, Baron C, Lobell DB (2017). Assessing climate adaptation options and uncertainties for cereal systems in West Africa. Agr. Forest Meteorol..

[9] Sultan B (2013). Assessing climate change impacts on sorghum and millet yields in the Sudanian and Sahelian savannas of West Africa. Environmental Research Letters.

[10] Nelson, G. C. *et al*. The Costs of Agricultural Adaptation to Climate Change. *The World Bank*, *Discussion Paper* **4**, http://siteresources.worldbank.org/EXTCC/Resources/407863-1229101582229/D%26CCDP_4-Agriculture9-15-10.pdf (accessed 29 January, 2018) (2010).

[11] Ignaciuk, A. & Mason-D’Croz, D. Modelling Adaptation to Climate Change in Agriculture, OECD *Food*, *Agriculture and Fisheries Papers* **70**, OECD Publishing, 10.1787/5jxrclljnbxq-en (2014).

[12] Iizumi T (2018). Crop production losses associated with anthropogenic climate change for 1981–2010 compared with preindustrial levels. International Journal of Climatology.

[13] Mizuta R (2016). Over 5000 years of ensemble future climate simulations by 60 km global and 20 km regional atmospheric models. Bull. Amer. Meteor. Soc..

[14] Shiogama H (2016). Attributing historical changes in probabilities of record-breaking daily temperature and precipitation extreme events. SOLA.

[15] Imada Y (2017). Recent enhanced seasonal temperature contrast in Japan from large ensemble high-resolution climate simulations. Atmosphere.

[16] Harris I, Jones PD, Osborn TJ, Lister DH (2014). Updated high-resolution grids of monthly climatic observations - the CRU TS3.10 Dataset. International Journal of Climatology.

[17] Iizumi T, Takikawa H, Hirabayashi Y, Hanasaki N, Nishimori M (2017). Contributions of different bias-correction methods and reference meteorological forcing data sets to uncertainty in projected temperature and precipitation extremes. Journal of Geophysical Research-Atmospheres.

[18] Rowell, D. *et al*. Initial Lists of AMMA-2050 User-Relevant Climate Metrics. AMMA-2050 *Technical Report* **1**, available from: www.amma2050.org (2015).

[19] Craufurd PQ, Vadez V, Jagadish SVK, Prasad PVV, Zaman-Allah M (2013). Crop science experiments designed to inform crop modeling. Agric. For. Meteorol..

[20] White JW, Hoogenboom G, Kimball B, Wall GW (2011). Methodologies for simulating impacts of climate change on crop production. Food Crop. Res..

[21] Baron C (2005). From GCM grid cell to agricultural plot: scale issues affecting modelling of climate impact. Philosophical Transactions of the Royal Society B: Biological Sciences.

[22] Kouressy M, Dingkuhn M, Vaksmann M, Heinemann AB (2008). Adaptation to diverse semi-arid environments of sorghum genotypes having different plant type and sensitivity to photoperiod. Agric. For. Meteorol..

[23] Mishra A (2008). Sorghum yield prediction from seasonal rainfall forecasts in Burkina Faso. Agric. For. Meteorol..

[24] Sinclair TR, Ludlow MM (1986). Influence if soil water supply on the plant water balance of four tropical grain legumes. Aust. J. Plant Physiol..

[25] Traoré SB (2011). Characterizing and modeling the diversity of cropping situations under climatic constraints in West. Africa Atmos. Sci. Lett..

[26] Monfreda C, Ramankutty N, Foley JA (2008). Farming the planet: 2. Geographic distribution of crop areas, yields, physiological types, and net primary production in the year 2000. Global Biogeochem. Cycles.

[27] Mueller ND (2012). Closing yield gaps through nutrient and water management. Nature.

[28] Marteau R (2011). The onset of the rainy season and farmers’ sowing strategy for pearl millet cultivation in Southwest Niger. Agric. For. Meteorol..

[29] Portmann FT, Siebert S, Döll P (2010). MIRCA2000—Global monthly irrigated and rainfed crop areas around the year 2000: A new high-resolution data set for agricultural and hydrological modeling, *Global Biogeochem*. Cycles.

[30] Siebert S (2015). A global data set of the extent of irrigated land from 1900 to 2005. Hydrol. Earth Syst. Sci..

[31] Allen RG, Pereira LS, Raes D, Smith M (1998). Crop evapotranspiration - *Guidelines for computing crop water requirements-FAO Irrigation and drainage paper***56**. Fao, Rome.

[32] Salack, S. *Impacts des Changements Climatiques sur la Production du Mil et du Sorgho Dans Les Sites Pilotes du Plateau Central*, *de Tahoua et de Fakara*. Niamey: CILSS (2006).

[33] Schlenker W, Lobell DB (2010). Robust negative impacts of climate change on African agriculture. Environ. Res. Lett..

[34] Cook KH, Vizy EK (2015). Detection and analysis of an amplified warming of the Sahara Desert. Journal of Climate.

[35] Nicholson SE, Funk C, Fink AH (2017). Rainfall over the African continent from the 19th through the 21st century. Global and Planetary Change.

[36] Taylor CM (2017). Frequency of extreme Sahelian storms tripled since 1982 in satellite observations. Nature.

[37] Panthou G, Vischel T, Lebel T (2014). Recent trends in the regime of extreme rainfall in the Central Sahel. Int. J. Climatol..

[38] Berg A, de Noblet-Ducoudre N, Sultan B, Lengaigne M, Guimberteau M (2013). Projections of climate change impacts on potential C4 crop productivity over tropical regions. Agric. For. Meteorol..

[39] Sultan B, Guan K, Kouressy M, Biasutti M, Piani C, Hammer G L, McLean G, Lobell D B (2014). Robust features of future climate change impacts on sorghum yields in West Africa. Environmental Research Letters.

[40] Challinor AJ (2014). A meta- analysis of crop yield under climate change and adaptation. Nat. Clim. Change.

