# Supplementary material for: Evidence of crop production losses in West Africa due to historical global warming in two crop models
Source: Sci Rep. 2019 Sep 6;9:12834. doi: 10.1038/s41598-019-49167-0 (PMC6731230; doi:10.1038/s41598-019-49167-0)
Supplement: Supplementary file 1 — Supplementary Information [file 41598_2019_49167_MOESM1_ESM.pdf]

Evidence of crop production losses in West Africa due to historical global warming in two crop models

Supplementary Information

Benjamin Sultan [1], Dimitri Defrance [1], and Toshichika Iizumi [2]

[1] ESPACE-DEV, Univ Montpellier, IRD, Univ Guyane, Univ Reunion, Univ Antilles, Univ Avignon, Maison de la Télédétection, 500 rue Jean-François Breton, F-34093 Montpellier Cedex, France
[2] Institute for Agro-Environmental Sciences, National Agriculture and Food Research Organization, 3-1-3 Kannondai, Tsukuba, Ibaraki 305-8604 Japan

Supplementary text

Sensitivity experiments using CYGMA model. We compared six types of CYGMA model simulation to characterize the sensitivity of modeled yield to individual factors. The sensitivity experiments include: (1) "no_co2" run, CO2 fertilization effect was removed in the model by using the constant CO2 level of 285 ppm (that is, the year 1850 level) as the input; (2) "no_tech" run, technological effect represented by increased use of N fertilizer and improved varieties that are more tolerant to suboptimal growing conditions than traditional ones in the model was removed by using the constant N fertilizer and agricultural knowledge stock levels of the year 2005 as the inputs; (3) "no_heat" run, heat stress reducing daily increment of leaf area and yield in the model was deactivated by modifying the model code; and (4) "no_wdef" run, water deficit stress in the model was deactivated in the similar manner with the no_heat run. In addition, (5) "subset" run based on the subset (25 members, out of 100) used for the sensitivity experiments mentioned above was presented. A use of all ensemble members for the sensitivity experiment is too computationally heavy to carry out. The subset run served as the reference. We also compared the subset run with (6) "full" run based on the all 100 ensemble members (that is, the data is presented in main text) to check if the subset run can represent the average estimate of the full run. The simulated grid-cell yields were aggregated to calculate average yield over West Africa and further averaged over the 2000-2009 period. The regional decadal average yields were used throughout the sensitivity experiments.
In the model, the CO2 fertilization effect always leads to yield gains by increasing the radiation-use efficiency. The deactivation of heat stress or water deficit stress always increases modeled yields, although the amplitude of yield gains varies depending on which heat or water deficit more strongly limits modeled yields in a given region. The removal of technological effect in the model could increase or decrease modeled yields. In the model, the crops' tolerance to heat and water deficit stresses (and cold and water excess stresses) changes with the agricultural knowledge stock. The relationship between the tolerance to the individual abiotic stresses and knowledge stock is determined using the global crop and climate datasets in 2000 to explain spatial variations in yields across countries, as elaborated in Iizumi et al. (2017). The modeled tolerance could increase when a region of interest locates in developing countries, of which knowledge stock is below the global average, and the technological effect is deactivated.

The sensitivity experiment results are shown in Fig. S6. The average estimates of the subset run were comparable to those of the full run. When the CO2 fertilization was deactivated, the modeled yields of millet and sorghum under the factual climate condition decreased, relative to the reference (the subset run) (the no_co2 run in Fig. S6 a and b). This indicates the benefit of the increased CO2 for the modeled yields. However, as expected, the CO2 fertilization effect did not appear in the counterfactual climate condition (the no_co2 run in Fig. S6 c and d). The deactivation of heat stress or water deficit stress increased yields with varying amplitude by crop, but the tendency that water deficit stress is a main yield limiting factor compared to heat stress is almost always consistent across the crops and across the factual and counterfactual climate conditions (the no_heat run and no_wdef

run in Fig. S6 a, b, c and d). In West Africa, the consideration of the knowledge stock decreased the modeled tolerance to stress associated with heat and water deficit, compared to the global average level. The removal of the technological effect increased the modeled tolerance and subsequent yields regardless of crops and climate conditions (the no_tech run in Fig. S6 a, b, c and d). The yield increases found in the no_tech run (relative to the reference) in the counterfactual climate condition were larger than those in the factual climate condition. The shortened crop duration due to warming and many more heat episodes in the factual climate condition than in the counterfactual climate condition may explain the smaller yield increases under the factual climate condition in the no_tech run.

The difference between the modeled yields under the factual and counterfactual climate conditions corresponds to the yield impact associated with historical climate change discussed in main text (Fig. S6 e and f). The subset run was able to capture the average estimate of yield impact computed from the full run, although the subset run showed relatively severer impact than the full run. The use of a small number of ensemble members is the reason for the discrepancy. The no_wdef run and no_heat run showed the smallest and severest yield impacts, respectively (Fig. S6 e and f). The negative yield impacts found in the no_wdef run was attributed to heat stress that was considered in the no_wdef run but not in the no_heat run. In contrast, the negative yield impacts appeared in the no_heat run was attributed to water deficit stress that was considered in the no_heat run but not in the no_wdef run. Note that the CO2 fertilization effect and technological effect were commonly considered in the no_heat run and no_wdef run (and cold stress and water excess stress in the studies region were negligibly small). These results suggest that water deficit stress is the major cause of the yield impacts associated with historical climate change. However, no significant trend in growing season rainfall was detected (Fig. 7). Hence, increases in evapotranspiration led by warming and associated water deficit stress are a likely underlying mechanism. The finding mentioned earlier that water deficit is a main limiting factor of the modeled yields in the studied region is consistent with this thought. The yield impact computed from the no_co2 run was severer than that calculated from the reference (the no_co2 run in Fig. S6 e and f). This is simply because that in the no_co2 run the modeled yields in the factual climate condition was lower than those in the counterfactual climate condition (the no_co2 run in Fig. S6 a, b, c and d). Also note that both heat stress and water deficit stress were considered in the no_co2 run. The same explanations are applied to the no_tech run showing the severer yield impacts than the reference.

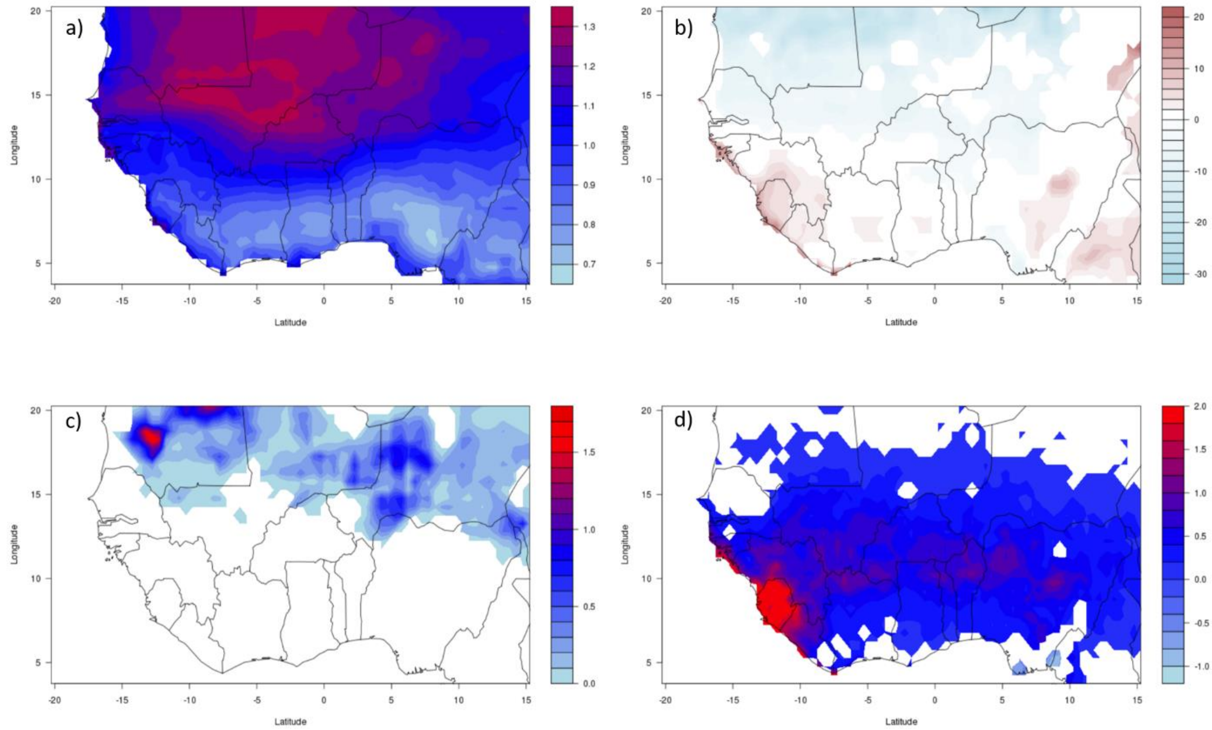

Fig. S1. Geographical patterns of climate indices changes in 2000–2009 associated with historical climate change, relative to a counterfactual climate condition. Shaded values represent the 100-members mean difference between the simulated factual and counterfactual climate conditions of mean surface temperature (a; in °C), annual rainfall (b; in %), number of very hot days (c; in day) and number of very heavy rainy days (d; in day). White values indicate the change is non-significant at the 1% level.

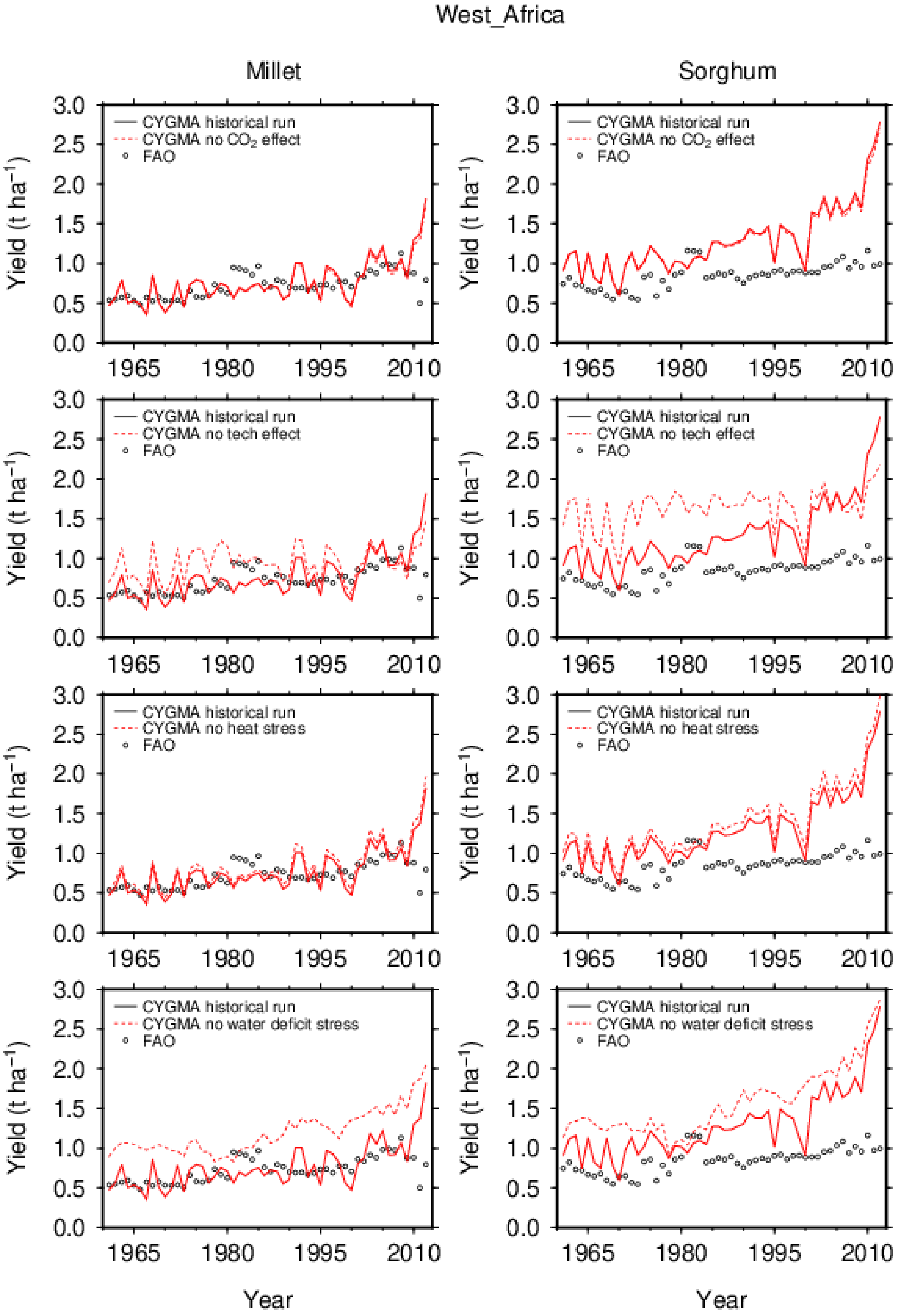

Fig. S2. Sensitivity analysis results for CYGMA using S14FD. Four runs were conducted (no CO2 effect run, no technological improvement effect run, no heat stress run and no water deficit stress run). The historical run presented in main text (Fig.3) was used as the reference. The sensitivity experiments

using CYGMA model revealed that technological improvement and water deficit respectively contributes to increase and decrease the simulated yield of the crops and these factors are followed by heat stress and to lesser extent CO2 effect. The CO2 effect increasing the simulated yield is tiny for the crops mainly because the difference in CO2 concentration of about 100 ppm between the two runs (285 ppm in the no CO2 run and 389 ppm in 2010 in the historical run) is small, and millet and sorghum are C4 crops. The simulated yield trend of the crops became less prominent when the technological level used in CYGMA model (that is, increased use of N fertilizer and improved varieties) was fixed to be its year 2005 level throughout the historical period (the no tech run). The simulated yields increased when either of heat stress or water deficit stress was omitted in the model (the no heat stress run and the no water deficit stress run). Water deficit appeared to be more dominant stress factor than heat for the simulated historical yield in the studied region. See **Supplementary text** for more details on the sensitivity experiments.

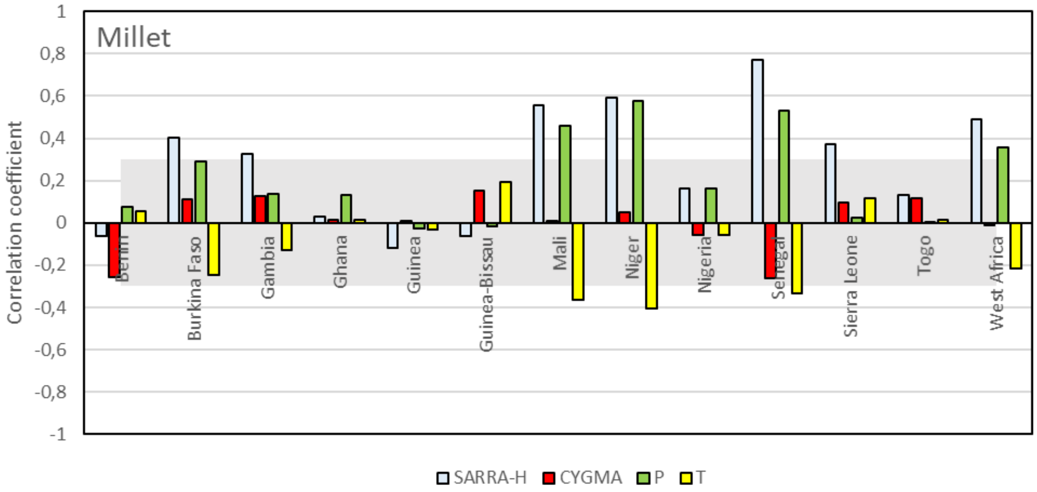

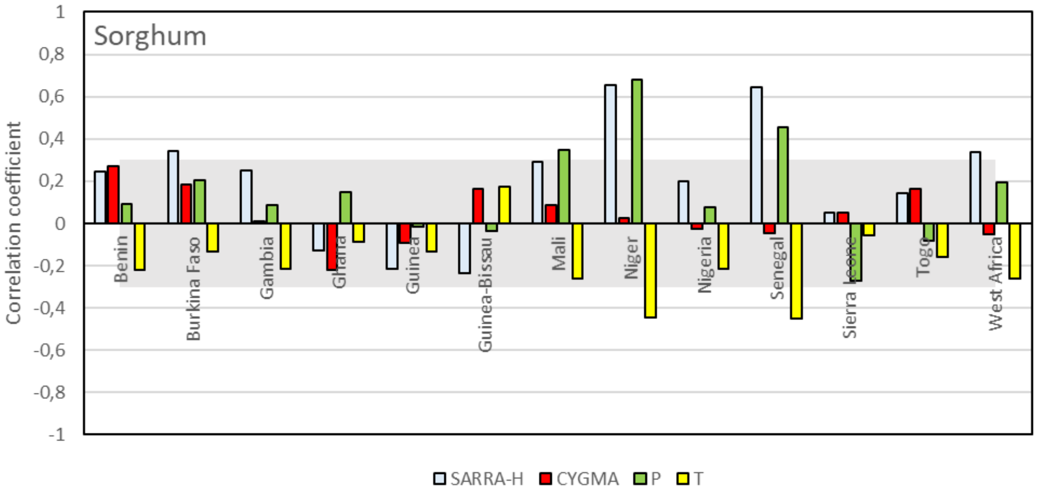

Fig. S3: Correlation coefficients between yield anomalies time series of millet (top) and sorghum (bottom) reported by FAO and simulated crop yield anomalies (SARRA-H and CYGMA) and growing season average temperature (yellow) and precipitation (green). Correlations were computed using simulated crop yield anomalies from SARRA-H and CYGMA, reported FAO yield anomalies and climate indices from S14FD for the period 1961-2012 at the country-level. The yield anomalies, relative to the normal yield calculated as the 5-yr running average, were separately computed for the reported and simulated data. The regional averaged data for West Africa were calculated by averaging the data over Benin, Burkina Faso, Gambia, Guinea, Guinea Bissau, Mali, Niger, Nigeria, Senegal, Sierra Leone and Togo (country harvested areas were used as the weights). The grid-cell harvested area in 2000 was used as the weights when computing country average yields. Values outside the grey shaded area are significant at the 5% level of confidence.

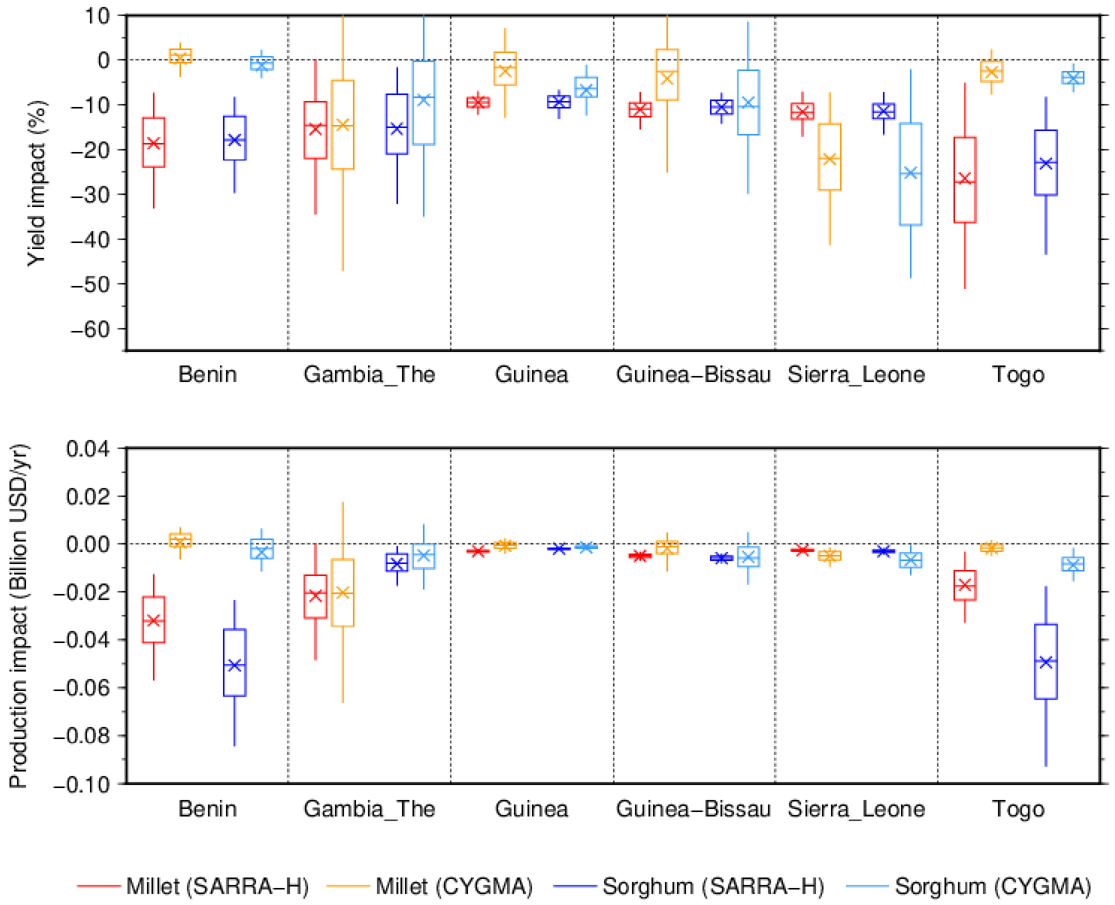

Fig. S4. Estimated impacts on average annual yield and average production of millet and sorghum in 2000–2009 for crop-producing countries in West Africa. The impacts were measured as the difference between the factual and counterfactual crop simulations.

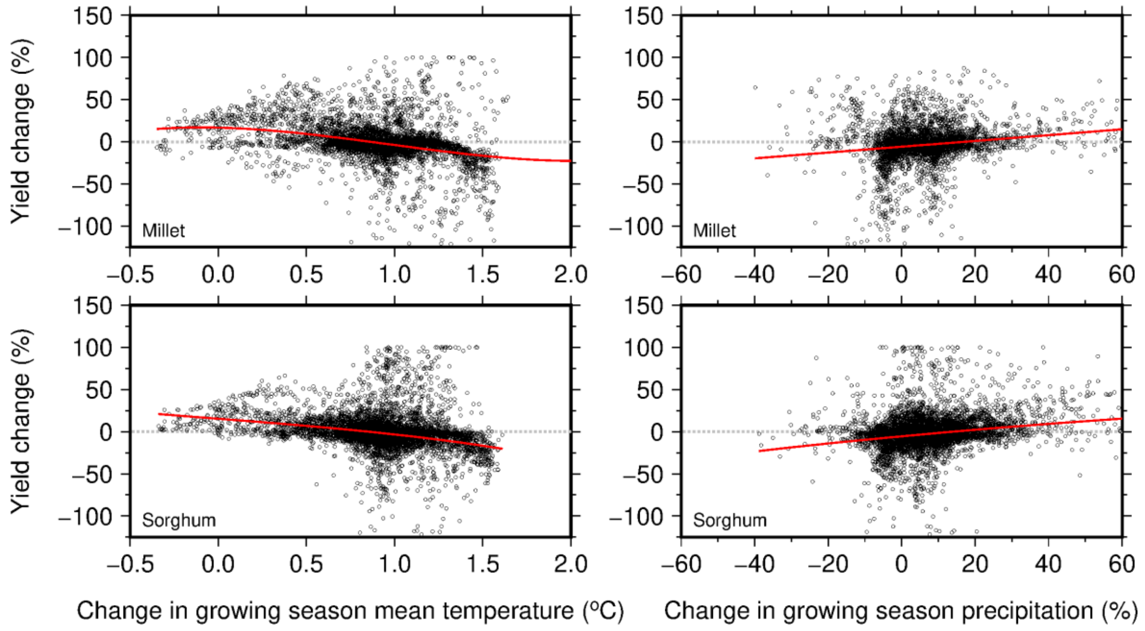

Fig. S5. Relative yield changes of millet and sorghum simulated by CYGMA in 2000–2009, relative to a counterfactual climate condition and relative changes of rainfall (right) and absolute change of mean surface temperature (left). A dot indicates individual grid cell. Average data over the 100 members are resented. Red lines indicate the regression curve that provides the best fit to the data in terms of the Akaike information criterion (AIC) by comparing four regression models (intercept-only, linear, quadratic and cubic models). The coefficients of determination (r^2) and p values are as follows: (upper left) millet versus temperature, r^2=0.073 ($p<0.001$); (upper light) millet versus precipitation, r^2=0.027 ($p<0.001$); (lower left) sorghum and temperature, r^2=0.019 ($p<0.001$); and (lower right) sorghum and precipitation, r^2=0.027 ($p<0.001$).

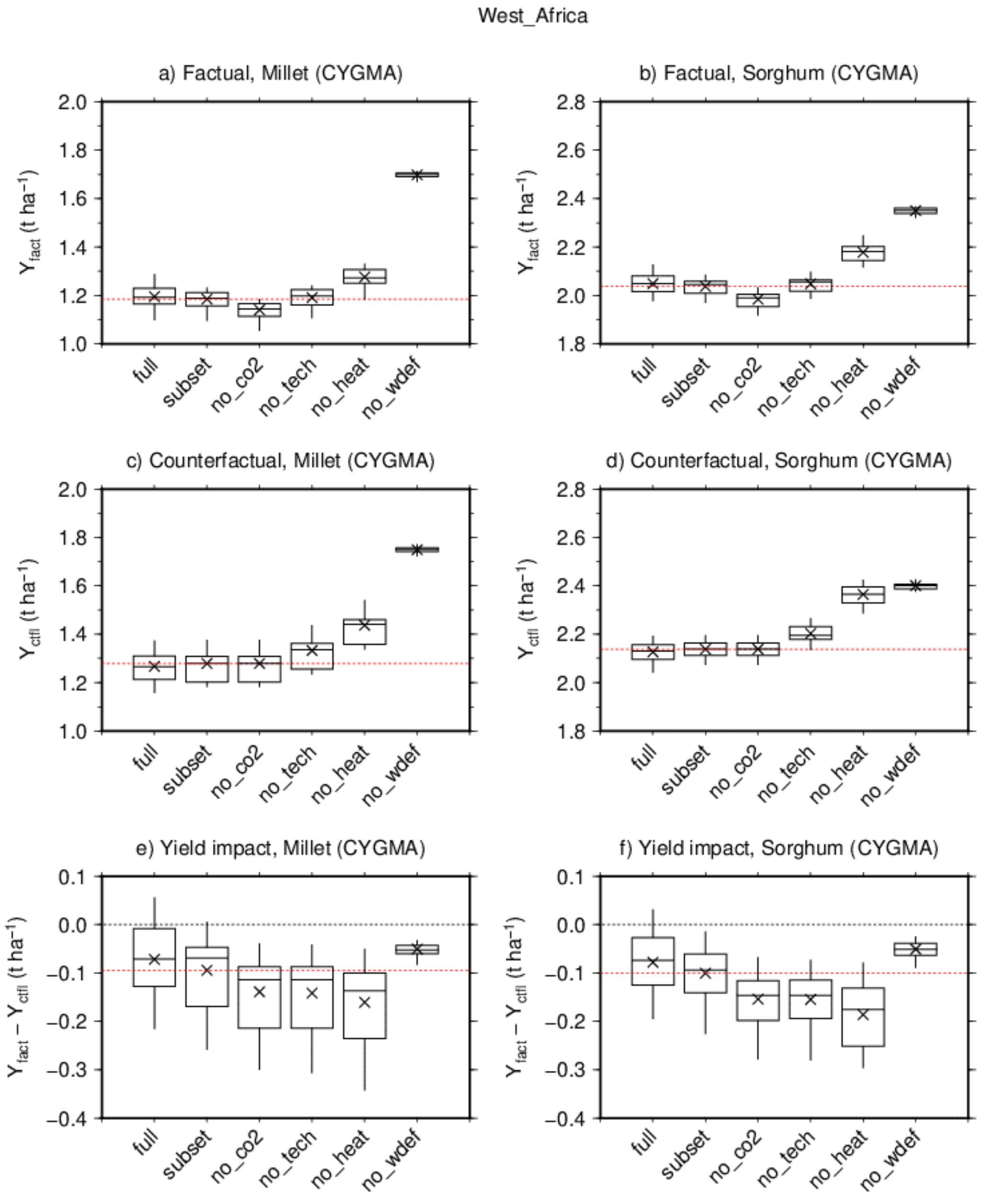

Fig. S6. The sensitivity of CYGMA-simulated regional decadal average yield of millet and sorghum for West Africa in 2000-2009 to individual factors. "full" indicates the 100-member crop simulation presented in main text; "subset" is based on the selected 25 members (out of 100) used to the sensitivity experiment and used as the reference (its average estimate is indicated by red line); "no_co2" indicates that CO2 fertilization in the model is deactivated; "no_tech" indicates that the modeled technological effect is removed; and "no_heat" and "no_wdef" indicates that heat stress and water deficit stress in the model was deactivated, respectively. Box plots indicate the mean (cross), with 25 to 75% (box) and 5 to 95% (vertical line) confidence intervals derived from the 25 members (100 members for the full run). The horizontal lines indicate the median.

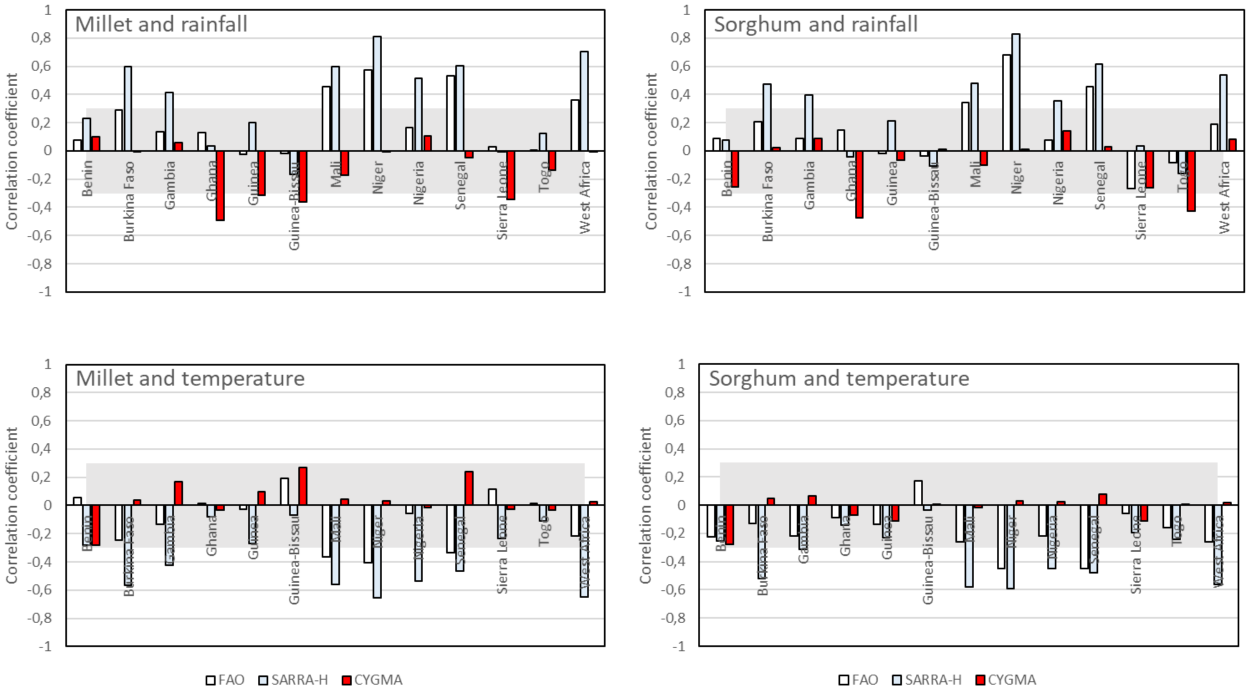

Fig. S7. Correlation coefficients between crop yield of millet and sorghum and growing season average temperature (top) and precipitation (bottom). Correlations were computed using simulated crop yield anomalies from SARRA-H and CYGMA, reported FAO yield and climate indices from S14FD for the period 1961-2012 at the country-level. The yield anomalies, relative to the normal yield calculated as the 5-yr running average, were separately computed for the reported and simulated data. The regional averaged data for West Africa were calculated by averaging the data over Benin, Burkina Faso, Gambia, Guinea, Guinea Bissau, Mali, Niger, Nigeria, Senegal, Sierra Leone and Togo (country harvested areas were used as the weights). The grid-cell harvested area in 2000 was used as the weights when computing country average yields. Values outside the grey shaded area are significant at the 5% level of confidence.

Table S1. Crop coefficients values for millet and sorghum used in this study. See also Supplementary Table S4 in Iizumi et al. (2017a) for more details.

Variable	Millet	Sorghum	Reference(s)
a (-)	I[1]: 4140, R[2]: 3814	I: 3623, R: 3623	This work. The method described in Iizumi et al. [6] was used.
b (-)	I: 14204, R: 11938	I: 12458, R:12458	
T_b (°C)	10	11	Neitsch et al. (2005)
T_u (°C)	30	30	
T_o (°C)	40	40	
$fr_{GDD, sen}$ (-)	0.6	0.6	
LAI_{max} (m^2 m^{-2})	2.5	3.	
l_1 (-)	3.055	3.055	
l_2 (-)	13.385	13.385	
h_{max} (m)	0.85	1.0	
p_1 (-)	12.915	12.915	
p_2 (-)	25.433	25.433	
$fr_{GDD, ant}$ (-)	0.482	0.482	
k (-)	0.65	0.65	
r_1 (-)	5.939	5.902	
r_2 (-)	-0.001	-0.002	
$Napp_o$ (kg N ha^{-1} yr^{-1})	210	210	The same values with maize were used (see Iizumi et al. [6] for details).
$Napp_{min}$ (kg N ha^{-1} yr^{-1})	-550	-550	
f_{Ndef} (-)	-0.0264	-0.0264	
g_{Ndef} (-)	10.6629	10.6629	
f_{heat} (-)	-0.0025	-0.0025	
g_{heat} (-)	0.1689	0.1689	
f_{cold} (-)	-0.0025	-0.0025	
g_{cold} (-)	0.3190	0.3190	
f_{Wdef} (-)	-0.0025	-0.0025	
g_{Wdef} (-)	0.1496	0.1496	
f_{Wexs} (-)	-0.00025	-0.00025	
g_{Wexs} (-)	0.04579	0.04579	

[1] Irrigated condition.

[2] Rainfed condition.

Table S2. Total production of millet and sorghum for West Africa in 2016 and production shares by country. The data were obtained from FAO statistical database. Characters placed in the right of data indicate the quality of data: "I" indicates that the data imputation was conducted by FAO; "U" indicates that the data were taken from unofficial sources; and if no character is shown, the data were taken from official sources. "n.a." indicates data are not available.

	Millet		Sorghum	
Total production in 2016 (Mt)	9.4		12.9	
	Share (%)			
Benin	0.3		1.0	
Burkina Faso	11.2	I	13.5	I
Cabo Verde	n.a.	n.a.	n.a.	n.a.
Côte d'Ivoire	0.6	I	0.4	I
Gambia	1.1	I	0.2	I
Ghana	1.7		1.8	
Guinea	2.1	I	0.3	I
Guinea-Bissau	0.1	U	0.1	U
Mali	19.1		10.8	
Mauritania	0.0	I	0.6	I
Niger	41.1		14.0	
Nigeria	15.6	I	53.7	I
Senegal	6.5	I	1.3	I
Sierra Leone	0.4	I	0.2	I
Togo	0.3		2.1	
